# Is *Stent* *Re*traction to Re*L*ieve *A*rterial *C*erebral Va*S*ospasm Caused by *S*AH (Stent-ReLACSS) Using PRELAX the Long-awaited Solution for Treatment of Posthemorrhagic Cerebral Vasospasm? Treatment of Posthemorrhagic Cerebral Vasospasm with PRESET and PRELAX: Technical Aspects, Efficacy, and Safety Margins in a Case Series

**DOI:** 10.1007/s00062-024-01402-6

**Published:** 2024-04-18

**Authors:** A. Khanafer, P. von Gottberg, P. Albiña-Palmarola, T. Liebig, M. Forsting, O. Ganslandt, H. Henkes

**Affiliations:** 1 Neuroradiologische Klinik, Neurozentrum, Klinikum Stuttgart, Stuttgart, Germany; 2 Department of Neuroradiology, University Hospital Munich (LMU), Munich, Germany; 3 Medizinische Fakultät, Universität Duisburg-Essen, Essen, Germany; 4 Neurochirurgische Klinik, Neurozentrum, Klinikum Stuttgart, Stuttgart, Germany

**Keywords:** Subarachnoid hemorrhage, Posthemorrhagic cerebral vasospasm, Endovascular treatment, Self-expanding stent, pRELAX

## Abstract

**Purpose:**

Recent observational studies have indicated the efficacy of stent retriever devices for the treatment of posthemorrhagic cerebral vasospasm (CVS), both by deployment and on-site withdrawal into the microcatheter (stent angioplasty, SA) and deployment followed by retraction through the target vessel similar to thrombectomy (*Stent* *Re*traction to re*L*ieve *A*rterial *C*erebral va*S*ospasm caused by *S*AH, *Stent-ReLACSS*). This article reports the findings with each application of pRESET and pRELAX in the treatment of CVS.

**Methods:**

We retrospectively enrolled 25 patients with severe CVS following aneurysmal subarachnoid hemorrhage. For the SA group, a stent retriever or a pRELAX was temporarily deployed into a narrow vessel segment and retrieved into the microcatheter after 3 min. For the Stent-ReLACSS group, a pRELAX was temporarily deployed into a narrow vessel and pulled back unfolded into the internal carotid artery. If intra-arterial vasodilators were administered, they were given exclusively after mechanical vasospasmolysis to maximize the effectiveness of the stent treatment.

**Results:**

In this study fifteen patients and 49 vessels were treated with SA. All were technically successful without periprocedural complications; however, 8/15 patients (53.3%) required additional treatment of the CVS. A total of 10 patients and 23 vessel segments were treated with Stent-ReLACSS. All maneuvers were technically successful without periprocedural complications and all vessels showed significant angiographic improvement. No recurrent CVS requiring further endovascular treatment occurred in-hospital, and neither territorial ischemia in the treated vessels nor vascular injury were observed in follow-up angiography.

**Conclusion:**

Based on the presented data it appears that Stent-ReLACSS with pRELAX does not pose any additional risks when used to treat CVS and might be superior to SA, especially concerning mid-term and long-term efficacy. The mechanism of action may be an effect on the endothelium rather than mechanical vasodilation. As many patients with CVS are diagnosed too late, prophylactic treatment of high-risk patients (e.g., poor grade, young, female) is potentially viable.

## Introduction

Over the past 20 years there have been increasing reports of various causes of delayed cerebral ischemia (DCI) without radiographic evidence of cerebral vasospasm (CVS). These studies described several phenomena, such as the development of cytotoxic and vasogenic edema due to spreading depolarization after aSAH [1, 2], microvascular dysfunction and microthrombosis due to distal cerebral vasospasm [3] and early brain injury due to the sudden increase in intracranial pressure [4]; however, CVS continues to be the leading cause of DCI and its associated delayed mortality and morbidity [5]. The occurrence of CVS after aneurysmal subarachnoid hemorrhage (aSAH) remains the leading cause of delayed mortality and neurological deterioration after treatment of ruptured aneurysms, despite the overall development of therapeutic and diagnostic measures [6, 7]. Vasospasm is radiologically detectable in 50–70% of patients with aSAH, of whom approximately 30% have neurological deficits [8, 9]. Conservative strategies after aSAH with intravenous (IV) or per os (PO) calcium channel blockers is standard in most neurovascular centers but it is often inadequate, especially in cases of severe and recurrent vasospasm [10]. Therefore, endovascular treatment (EVT) is applied in many cases. The treatment for CVS described in the literature includes medicinal angioplasty using intra-arterial (IA) vasodilators (e.g., nimodipine [11], milrinone [12, 13] and verapamil [14]), long-term intra-arterial infusion of nimodipine [15] and dilatation of large arteries with noncompliant balloons, compliant balloons [16, 17], stent retrievers [18–22] or by implantation of self-expending stents [23].

The pRELAX (WallabyPhenox, Bochum, Germany: femtos GmbH: Legal manufacturer, phenox GmbH: Sole distributor) is a dedicated device for the treatment of vasospasm. It was initially developed for stent angioplasty (SA) of vasospastic vessels. Unlike conventional stent retrievers, the pRELAX device provides consistently high radial force over its entire working length. Familiarity with the use of stent retrievers in mechanical thrombectomy has led to an increased use of these devices for the treatment of CVS, and several recent studies have demonstrated the beneficial effect of CVS treatment with angioplasty using self-expanding retrievable stents [18–22].

However, a significant number of patients with CVS are still not adequately treated. This article reports initial experiences with *Stent* *Re*traction to re*L*ieve *A*rterial *C*erebral va*S*ospasm caused by *S*AH (Stent-ReLACSS) using pRELAX and SA in the treatment of posthemorrhagic CVS.

## Methods

### Study Population

Between January 2010 and July 2023, a total of 1112 patients were treated for aSAH at the neurovascular center of Klinikum Stuttgart. For all patients, clinical data and the results of computed tomography (CT), digital subtraction angiography (DSA), and magnetic resonance imaging (MRI) were retrospectively evaluated. We identified 316 patients with angiographic CVS despite medicinal management including the routine IV administration of calcium antagonists.

### Management

The management strategy for aSAH patients is as follows:

- Early diagnosis of aSAH by CT, computed tomography angiography (CTA), or magnetic resonance imaging/angiography (MRI/MRA) followed by DSA and subsequently early endovascular or microsurgical aneurysm treatment after a multidisciplinary decision.
- Early external cerebrospinal fluid drainage (if deemed necessary).
- Administration of nimodipine IV or PO from the first clinical day of hospitalization (if tolerated).
- Close cardiovascular monitoring to avoid episodes of hypotension under CVS.
- Daily examination for CVS with transcranial doppler (TCD) and transcranial color doppler (TCCD) as well as periodic CTA and computed tomography perfusion (CTP) from the fourth day post-aSAH.
- Stellate ganglion blockade if incipient CVS is suspected.

A mean flow velocity (MFV) > 120 cm on TCD was considered as early or suspected CVS with the potential for progression. To confirm CVS, these patients subsequently underwent CT, CTA and CTP or MRI and MRA. If severe CVS was confirmed in CT or MRI, along with associated neurological deterioration in awake patients or associated hypoperfusion on CTP, all patients underwent DSA followed by EVT. We classified the vessel segment affected by CVS as moderate with a vessel diameter < 50% and severe with a vessel diameter of less than one third (approximately < 65%) that of the original vessel. After mechanical vasospasm treatment, we classified each vessel segment as adequately treated if the diameter of the treated vessel increased to more than two thirds (approximately 65%) of the normal diameter. Vessel diameters were measured with the calibrated three DSA systems on which the procedures were performed. For a long time, IA vasodilators were the first and standard treatment; however, due to the limited long-term effect and the challenging management of selective long-term IA infusion of nimodipine, we have increasingly used mechanical treatment. The two methods used for mechanical spasmolysis were stent angioplasty (SA) using a stent retriever or pRELAX device and the Stent-ReLACSS using pRELAX. At the beginning of our experience, we administered medicinal vasospasmolysis either before or during mechanical vasospasmolysis. As our experience grew, we exclusively administered drug spasmolysis after the mechanical treatment. All patients in both groups either did not receive drug vasospasmolysis or received it after mechanical vasospasmolysis.

### Stent Angioplasty Group

In this group, patients were treated using a pRELAX 4–20 mm or a stent retriever such as a pRESET 4–20 mm, pRESET 6–30 mm, or pRESET-LT 4–20 mm or 3–20 mm. The treatment was administered either at the first treatment session or at later sessions due to recurrent CVS. An IA vasospasmolysis was always performed after SA during the same treatment session.

A 0.021″ or 0.017″ inner diameter (ID) microcatheter was tracked to the narrow vessel segments over a microguidewire, after which a pRELAX or pRESET stent retriever (WallabyPhenox) was traced in the microcatheter and unsheathed. The device diameter was selected based on the original diameter of the affected vessels in the initial (prevasospasm) DSA.

The stent was deployed for approximately 3 min, after which it was resheathed into the microcatheter. After stent resheathing and IA vasospasmolysis, angiographic runs were performed to measure the degree of caliber change.

If CVS persisted, the stent retriever was redeployed a second time using the same method.

### Stent-ReLACSS

A 0.021″ ID microcatheter was advanced over a microguidewire to the narrow vessel segment, after which a pRELAX 4‑20 was advanced into the microcatheter and unsheathed.

**Fig. 1:**
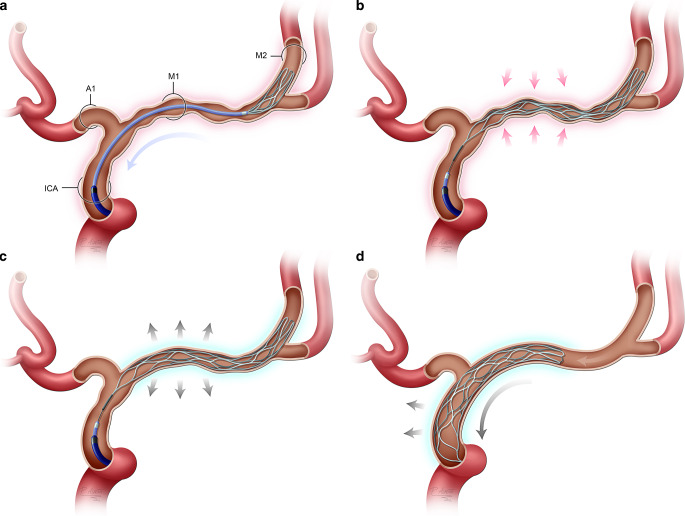
Diagram of the Stent-ReLACSS using pRELAX. **a** A 0.021″ microcatheter is inserted into the MCA (middle cerebral artery) or ICA (internal cerebral artery) segments affected by CVS using a microwire. The pRELAX device is inserted into the microcatheter, then the pRELAX device is released from the microcatheter without pushing to avoid vascular injury or aggravation of vasospasm. **b** The microcatheter is withdrawn over the wire of the pRELAX device into the extracranial segment of the ICA to avoid reduced perfusion of the vessels affected by the vasospasm. **c** After an incubation period of 3 min, some dilatation of the device and the corresponding vessel can be seen. **d** The pRELAX device is then pulled back into the guiding catheter in an open, fully deployed state, without resheathing, similar to mechanical thrombectomy

The stent was deployed for approximately 3 min, after which the pRELAX was pulled through the vessels affected by CVS, similar to mechanical thrombectomy. Angiographic examinations were performed immediately after the Stent-ReLACSS and again after 15 min. When measuring the degree of caliber change, the effect of the treatment could only be seen after 10–15 min. The Stent-ReLACSS using pRELAX was used exclusively in the intradural segment of the internal carotid artery (ICA) and in the M1 and M2 segments of the middle cerebral artery (MCA). Due to the lack of long-term experience with the pRELAX device, we did not use it in the anterior cerebral artery, posterior cerebral artery or basilar artery. For extended CVS, especially if the length exceeded 20 mm, the Stent-ReLACSS was performed twice, first between the M1 segment and the ICA and then between the M2 and M1 segments. There was no use of IA vasodilators or other EVT in any of the treated vessel segments before the Stent-ReLACSS. In a few cases, IA vasodilators were used after the Stent-ReLACSS to obtain a more rapid effect on the distal branches. To measure the degree of caliber change, angiographic runs were performed before IA vasospasmolysis (Fig. 1).

### Endovascular Treatment

All treatments were performed with the patient under general anesthesia using a 6F guiding catheter with guidance provided by biplane digital subtraction angiography (DSA) units (Axiom Artis, Siemens, Erlangen, Germany; Azurion, Philips, Eindhoven, The Netherlands). An intermediate catheter was not required. All patients received intravenous administration of 2000–3000 IU heparin. Heparinized irrigation saline solutions were used for all catheters (5000 IU unfractionated heparin/l).

### Follow-up

Early follow-up with catheter angiography was performed in most patients before discharge if possible, according to our routine clinical protocols. The first angiographic follow-up was performed 3–6 months after discharge in patients who survived.

## Results

### Stent Angioplasty Group

Stent angioplasty was performed in 15 patients with CVS after aSAH. Of these patients 11 were female and the mean age was 52.5 years (range 37–81 years). All 15 ruptured aneurysms were treated, 13 by EVT and 2 by neurosurgical clipping. A total of 49 narrow vessel segments were treated. In 7 of the patients it was the first treatment session; the treatments were performed on average 8.6 days after aSAH (range 4–14 days). The clinical and radiological characteristics of the patients are summarized in Table 1.

**Table 1:** Clinical and radiological findings of the patients

Patient number	Group	Sex	Initial HH	Fisher	Aneurysm localization	Aneurysm treatment	Days between SAH and vasospasm onset	Days between SAH and stent angioplasty
1	SA	F	5	4	ICA‑P	pCONUS + Coiling	7	8
2	SA	F	2	4	AcomA	Clipping	4	7
3	SA	F	3	4	ICA‑P	FD + Coiling	8	10
4	SA	F	3	4	ICA‑T	Clipping	6	6
5	SA	M	2	4	AcomA	Coiling	3	6
6	SA	F	2	3	AcomA	Coiling	8	8
7	SA	F	4	4	AcomA	Coiling	4	7
8	SA	M	2	4	MCA-bi	pCONUS + Coiling	13	13
9	SA	M	4	4	V4	FD	8	9
10	SA	F	4	4	PcomA	Coiling	12	13
11	SA	F	3	4	V4	FD	4	4
12	SA	F	2	3	PcomA	FD + Coiling	10	10
13	SA	M	5	4	PICA	Coiling	14	14
14	SA	F	3	4	BA	Coiling	6	7
15	SA	F	3	2	AcomA	Coiling	7	7
16	Stent-ReLACSS	F	1	1	ICA‑P	Coiling	5	11
17	Stent-ReLACSS	M	5	3	V4	FD	8	8
18	Stent-ReLACSS	M	3	4	PICA	Coiling	8	8
19	Stent-ReLACSS	F	3	3	MCA-bi	Coiling	11	11
20	Stent-ReLACSS	F	2	3	AcomA	Coiling	3	3
21	Stent-ReLACSS	F	5	4	AcomA	Clipping	9	10
22	Stent-ReLACSS	F	4	4	BA	Coiling	1	1
23	Stent-ReLACSS	M	4	4	ICA‑T	FD	3	6
24	Stent-ReLACSS	M	2	1	AcomA	Coiling	5	5
25	Stent-ReLACSS	M	1	1	AcomA	FD	10	10

*AcomA* anterior communicating artery, *BA* basilar artery, *F* female, *FD* flow diversion, *FD* *+* *Coiling* flow diversion assisted coiling, *HH* Hunt & Hess, *ICA‑P* posterior communicating artery, *ICA‑T* T of the internal carotid artery, *M* male, *MCA-bi* bifurcation of the middle cerebral artery, *pCONUS (WallabyPhenox, Bochum, Germany)* *+* *Coiling* pCONUS stent assisted coiling, *PICA* posterior inferior cerebellar artery, *SAH* subarachnoid hemorrhage, *V4* V4 segment of the vertebral artery

Of these segments 18 showed high-grade CVS with a narrowing of more than two thirds of the original vessel lumen, and 31 segments showed moderate CVS with a narrowing of more than 50% of the original vessel lumen. We performed SA on 2–9 vessel segments per patient. The 49 treated segments were ICA 2 segments, M1 12 segments, M2 8 segments, A1 18 segments, A2 5 segments, basilar artery (BA) 2 segments, and posterior cerebral artery (PCA) 2 segments. For stent angioplasty, we used the pRELAX 4‑20 device five times, the pRESET 4‑20 seven times, the pRESET-LT 4‑20 twice, and the pRESET 6‑30 once.

The SA was successful in 7/18 (38.9%) segments with high-grade CVS and in 20/31 (64.5%) segments with moderate-grade CVS. Overall, the 49 segments showed an average diameter improvement of 65.3% compared to the original vessel diameter, and in the 27 vessel segments where the treatment was successful, the average diameter improvement was 77.3%.

**Fig. 2:**
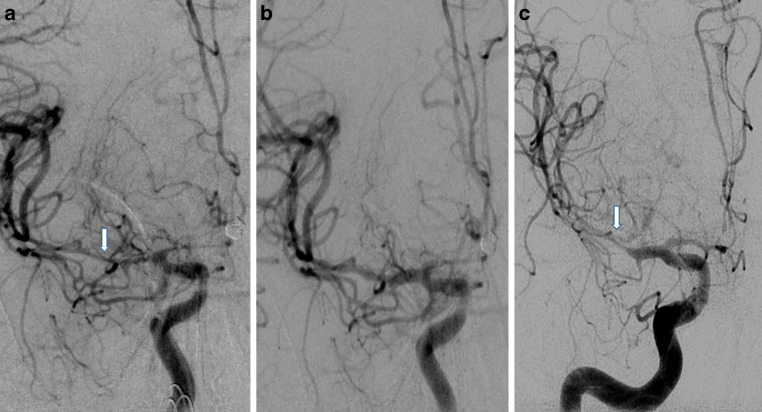
**a** Initial angiogram on day 5 after aSAH. Severe vasospasm of the right M1 and M2 segments (*arrow*). A pRELAX 4‑20 is deployed in the M1–M2 segment for 3 min. **b** Angiogram after completion of stent angioplasty using the pRELAX showing sufficient vasodilation. **c** Angiogram 1 day later showing recurrent vasospasm (*arrow*)

For all SA patients, we performed short-term intra-arterial medicinal treatment with a vasodilator in the same session. Of the 15 patients 8 (53.3%) required additional treatment of the recurrent or residual CVS segments, either in the same session (1/8) or in a later session (7/8) (Fig. 2). Subsequent treatments for recurrent CVS included bail out implantation of a self-expanding stent once at the same treatment session and long-term selective IA infusion of nimodipine in four other patients. Short-term IA milrinone was performed as a retreatment at later sessions in two patients and one patient underwent repeated SA. In total, 20/49 (40.8%) vessel segments had to be retreated, 1 in the same session and 19 in subsequent sessions. We did not observe any signs of vascular injury or abnormalities at early or late follow-up.

### Stent-ReLACSS

The Stent-ReLACSS was performed in 10 patients with CVS and 5 of the aSAH patients were male (mean age 48.1 years). One of the ruptured aneurysms was treated by neurosurgical clipping. A total of 24 narrow vessel segments were treated, 22 of which showed severe CVS with a narrowing of more than two thirds of the original vessel lumen, and 2 segment showed moderate CVS with a narrowing of more than 50% of the original vessel lumen. In five of the patients, Stent-ReLACSS was performed in the first treatment session. Treatments were performed on average 6.3 days after aSAH (range 1–11 days). The demographic and clinical characteristics of the patients are summarized in Table 1.

**Fig. 3:**
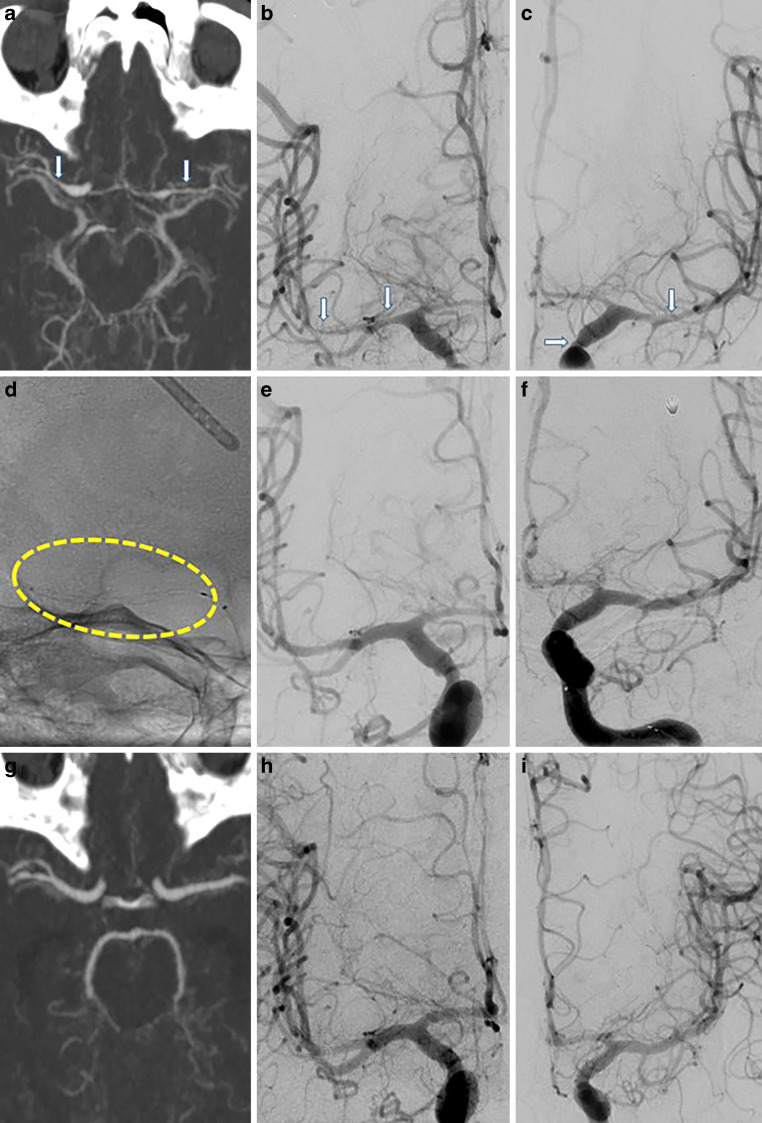
**a**, **b**, **c** Initial CTA (computed tomography angiography) and DSA (digital subtraction angiography) on post-ictus day 9. Vasospasm of M1 and M2 bilaterally (*arrows*). **d** pRELAX 4–20 mm deployed in M1–M2 segments. **e**, **f** DSA 15 min after Stent-ReLACSS (Stent Retraction to relieve Arterial Cerebral vasoSpasm caused by SAH) showing sufficient vasodilatation. **g** CTA 1 day after EVT (endovascular treatment) with Stent-ReLACSS showing sufficient vasodilatation. **h**, **i** Angiogram 15 days after Stent-ReLACSS showing regular vessel lumen and no vessel injury. No other endovascular treatment for CVS had been performed in the meantime for the internal cerebral artery or middle cerebral artery

After the Stent-ReLACSS, all vessel segments showed satisfactory dilatation and improved perfusion of the distal vasculature in both severely and moderately affected vessels. All vessel segments showed satisfactory vasodilation and improved perfusion of the distal vasculature after therapy. In 3 of the 10 patients, IA vasospasmolysis was performed after pRELAX RM, despite the success of mechanical vasospasmolysis, in order to achieve a more rapid effect on the distal vasculature. Overall, the 24 segments showed an average diameter improvement of approximately 83.4% compared with the original vessel diameter. None of the treated segments required repeated EVT at the same or subsequent session. No evidence of vascular injury or abnormality was observed at either early or late follow-up (Fig. 3).

**Fig. 4:**
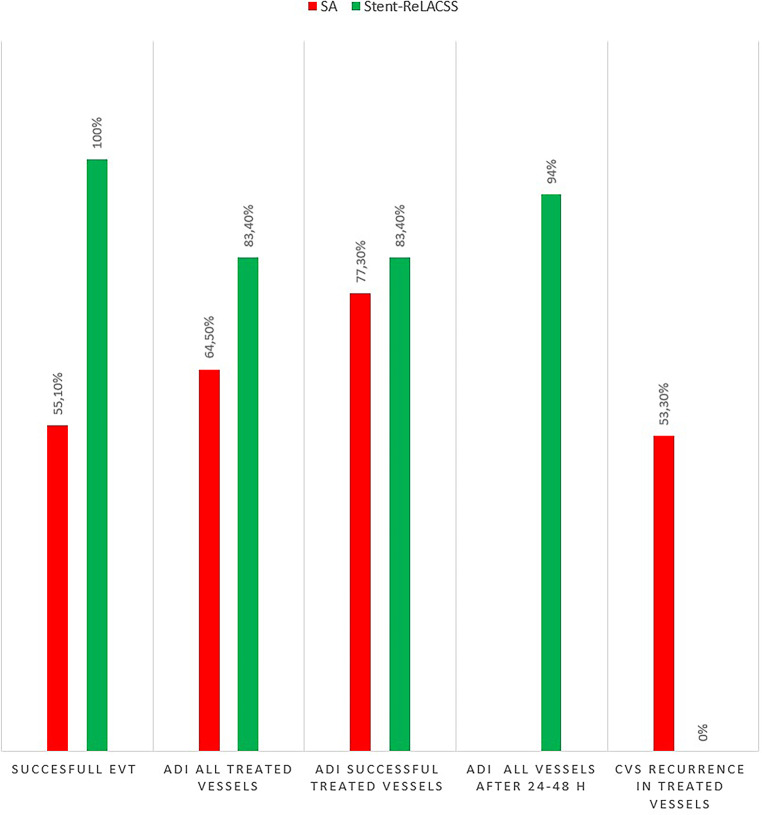
The bar graph displays the outcomes of endovascular treatments in both groups. *ADI* average diameter improvement, *CVS* cerebral vasospasm, *EVT* endovascular treatment, *H* hours, *SA* stent angioplasty, *Stent-ReLACSS* stent retraction to relieve arterial cerebral vasospasm caused by SAH

On examination of the early angiographic controls after the Stent-ReLACSS, we found that the full effect of the treatment was not seen until 24–48 h later. Overall, the vessels showed an average improvement in diameter of 94% after 24–48 h compared with 83.4% immediately after treatment. This effect of the maneuver on the diameter of the target vessels was documented through DSA and/or CTA. Out of the 10 patients 9 underwent DSA within the first 48 h, either as a follow-up or as part of CVS treatment of vessels in other territories. The CTA images of the remaining patient, who did not undergo a DSA follow-up, were compared. During the critical phase of vasospasm, CTA and CTP examinations were performed daily on the patients to observe the effect of the treatment. No retreatment of these vessel segments was necessary. The bar in Fig. 4 displays the outcomes of the endovascular treatments.

### Complications and Clinical Follow-up

We did not encounter any complications during or after EVT in either group. The patients’ mRS scores at discharge and first follow-up are summarized in Tables 2 and 3.

**Table 2:** Procedural details, radiological and clinical results and recurrence of the patients in Stent angioplasty group

Patient number	Total segments	Treated Segments	Grade of CVS	CVS relief	Device	Compli-cations	Total segments with recurrent CVS	Further EVT in the same session	EVT for recurrent CVS in later sessions	mRS on discharge	mRS at the first follow -up (months)
1	1	M1	1: severe	1: Insufficient	pRELAX 4‑20	No	1	Bail out implantation of a self-expanding stent	_	5	3 (6m)
2	2	A1	2: severe	1: Sufficient	pRELAX 4‑20	No	2	No	Long-term selective IA infusion of nimodipine	5	4 (3m)
		A2		1: Insufficient							
3	2	A1	1: severe	2: Sufficient	pRELAX 4‑20	No	0	No	Long-term selective IA infusion of nimodipine	5	4 (10m)
		A2	1: moderate								
4	2	M1	1: severe	2: Sufficient	pRELAX 4‑20	No	2	No	Short-term IA-milrinone	2	1 (3m)
		M2	1: moderate								
5	3	M1	2: severe	3: Sufficient	pRELAX 4‑20	No	1	No	Long-term selective IA infusion of nimodipine	5	4 (3m)
		A1	1: moderate								
		M2									
6	2	A1	2: moderate	2: Sufficient	pRESET-LT 4‑20	No	0	No	_	1	1 (7m)
		A2									
7	6	M1	2: severe	4: Sufficient	pRESET 4‑20	No	6	No	Long-term selective IA infusion of nimodipine	4	2 (3m)
		M2 both sides	4: moderate	2: Insufficient							
		A1 both sides									
		BA									
8	2	P1	1: severe	1: Sufficient	pRESET LT 4‑20	No	0	No	_	4	2 (4m)
		M2	1: moderate	1: Insufficient							
9	6	ICA	2: severe	4: Sufficient	pRESET 6‑30	No	1	No	Short-term IA-milrinone	4	4 (3m)
		M1 both sides	4: moderate	2: Insufficient							
		A1 both sides									
		BA									
10	3	ICA	3: moderate	3: Sufficient	pRESET 4‑20	No	0	No	_	5	4 (6m)
		M1									
		A1									
11	9	M1 both sides	2: severe	1: Sufficient	pRESET 4‑20	No	5	No	Short-term IA-milrinone + SA	2	1 (4m)
		M2 both sides	7: moderate	8: Insufficient							
		A1 both sides									
		A2 both sides									
		P1									
12	3	A1 both sides	3: moderate	2: Sufficient	pRESET 4‑20	No	2	No	Short-term IA milrinone	4	4 (7m)
		A2		1: Insufficient							
13	4	M1 both sides	2: severe	1: Sufficient	pRESET 4‑20	No	0	No	_	3	1 (1m)
		M2	2: moderate	3: Insufficient							
		A1									
14	2	M1	2: severe	2: Insufficient	pRESET 4‑20	No	0	No	_	6	6
		A1									
15	2	A1 both sides	2: moderate	2: Sufficient	pRESET 4‑20	No	0	No	_	3	3 (4m)

Sufficient if the diameter of target vessel is increased to more than 65% of normal diameter. Moderate CVS: luminal narrowing > 50%, severe CVS: luminal narrowing > 65%. Sufficient relief: increased to more than 65% of the normal diameter, insufficient relief: increased to less than 65% of the normal diameter

*A1* anterior cerebral artery A1 segment, *A2* anterior cerebral artery A2 segment, *BA* basilar artery, *CVS* cerebral vasospasm, *EVT* endovascular treatment, *ICA* internal carotid artery, *IA* intra-arterial, *m* months, *M1* middle cerebral artery M1 segments, *M2* middle cerebral artery M2 segments, *mRS* modified Rankin Score, *P1* posterior cerebral artery P1 segment, *SA* stent angioplasty

**Table 3:** Procedural details, radiological and clinical results and recurrence of the patients in Stent-ReLACSS using pRELAX group

Patient number	Total segments	Treated Segments	Grade of CVS	CVS relief	Device	Complications	Total segments with recurrent CVS	Further EVT in the same session	mRS on discharge	mRS at the first follow-up (months)	EVT for recurrent CVS in later sessions
1	3	ICA	3: severe	3: Insufficient	pRELAX 4‑20	No	0	No	6	6	No
		M1									
		M2									
2	4	ICA both sides	4: severe	4: Sufficient	pRELAX 4‑20	No	0	No	4	0 (8m)	No
		M1 both sides									
3	4	M1 both sides	4: severe	4: Sufficient	pRELAX 4‑20	No	0	No	4	1 (5m)	No
		M2 both sides									
4	2	M1	2: severe	2: Sufficient	pRELAX 4‑20	No	0	No	2	0 (3m)	No
		M2									
5	1	M1	1: severe	1: Sufficient	pRELAX 4‑20	No	0	No	4	2 (3m)	No
6	2	M1	2: severe	2: Sufficient	pRELAX 4‑20	No	0	No	5	5 (5m)	No
		M2									
7	4	ICA	4: severe	4: Sufficient	pRELAX 4‑20	No	0	No	6	6	No
		M1 both sides									
		M2									
8	1	M1	1: moderate	1: Sufficient	pRELAX 4‑20	No	0	No	4	3 (2m)	No
9	1	M1	1: moderate	1: Sufficient	pRELAX 4‑20	No	0	No	2	1 (4m)	No
10	2	ICA	2: severe	2: Sufficient	pRELAX 4‑20	No	0	No	2	1 (5m)	No
		M1									

Sufficient if the diameter of target vessel is increased to more than 65% of normal diameter. Moderate CVS: luminal narrowing > 50%, severe CVS: luminal narrowing > 65%. Sufficient relief: increased to more than 65% of the normal diameter, insufficient relief: increased to less than 65% of the normal diameter

*A1* anterior cerebral artery A1 segment, *A2* anterior cerebral artery A2 segment, *CVS* Cerebral vasospasm, *EVT* endovascular treatments, *ICA* internal carotid artery, *m* months, *M1* middle cerebral artery M1 segments, *M2* middle cerebral artery M2 segments, *mRS* modified Rankin Score

## Discussion

Since the 1970s, attempts have been made to treat CVS mainly with conservative strategies. Triple H treatment (hypertension, hypervolemia, and hemodilution) was systematically used to improve cerebral perfusion [24]. Increasing experience showed that only induced hypertension improved cerebral perfusion [25]: however, the effect of hypertension on DCI is now doubted [26]. Nimodipine is commonly administered to patients after aSAH either PO or IV to prevent vasospasm due to its vasodilatory effects, ability to cross the blood-brain barrier and neuroprotective properties [27–29]; however, these conservative measures are not sufficient to protect against CVS and the resulting DCI [10].

Recent studies have confirmed that early and aggressive treatment of CVS after aSAH protects against potential DCI and may thus improve outcomes [26, 30]. Short-term intra-arterial medicinal angioplasty with a vasodilator is the most commonly used EVT; however, it often shows only a transient effect [31, 32]. Infusion of vasodilators can lead to systemic hypotension and subsequent intracranial hypertension, especially when high doses are administered [32].

Long-term selective intra-arterial infusion of nimodipine is effective but this treatment requires an enormous logistic effort [15]. In addition, the required complete heparinization and prolonged ventilation time may lead to severe hemodynamic and respiratory complications.

Several studies over the last 10 years have highlighted the beneficial effects of mechanical vasospasmolysis in the treatment of severe cerebral vasospasms. To date, transluminal balloon angioplasty is the most commonly used treatment for mechanical vasospasmolysis in CVS and has been shown to be an effective EVT, providing rapid and sustained improvement in vessels affected by CVS [11, 12]. In their international multicenter online survey, Guenego et al. found that 79% (161/201) of the participating physicians (endovascular neurosurgeons, neuroradiologists, and neurologists) preferred IA vasospasmolysis with medication as the first choice for EVT, while only 14% (31/201) used mechanical vasospasmolysis as the initial treatment method; however, only 17% (35/201) of users reported that medication-induced IA vasospasmolysis is consistently effective in more than 75% of cases. In comparison, 48% (97/201) considered balloon angioplasty-based mechanical vasospasmolysis to be effective in more than 75% of cases, while only 14% (28/201) considered it to be effective in 50% or less of cases. Additionally, 65% of neurointerventionalists believed that mechanical angioplasty is the most effective endovascular treatment [33]; however, transluminal balloon angioplasty has serious limitations in the treatment of distal or elongated vessels [33, 34]. In addition, balloon angioplasty procedures may cause vessel injury, such as arterial dissection or rupture as well as ischemic insults due to flow interruption [34–36].

In recent years, several studies have demonstrated the safety and efficacy of CVS treatment by angioplasty using stent retrievers [18–22]**.** Bhogal et al. first described treatment using the pRESET (Wallaby Phenox) in patients with iatrogenic vasospasm by surgical manipulation [20].

Kwon et al. compared the effect of stent retriever angioplasty before and after vasodilator administration. In the vasodilator-first group, 71.4% of the treated segments (10/14) showed vasodilation after stentoplasty but 60% of the patients (3/5) developed recurrent vasospasm requiring repeated angioplasty. In the stent retriever-first group, 82.1% of the segments (32/39) showed vasodilation after stentoplasty, but none of the patients developed radiologic or clinical signs of recurrent vasospasm [18]. The authors concluded that the use of stent retrievers for the treatment of CVS can result in long-term vasodilatation, especially when the stent retriever is used before infusion of IA vasodilators.

Su et al. reported the successful use of a Solitaire (6/40 mm; Medtronic, Irvine, CA, USA) stent retriever in distal vessels suffering from CVS, such as A2, M2, and P2 segments [22]. In the study six patients with CVS after aSAH showed recurrent vasospasm despite administration of IA vasodilators. The stent retriever was deployed for 2–5 min in 14 vasospastic vessel segments of 6 patients. Subsequently, all patients showed radiologic resolution of CVS.

Khanafer et al. described the first treatment of CVS with self-expanding stent deployment, resulting in long-term vasodilation; however, this treatment is a bail-out option that is not desirable for all CVS patients [23].

These published studies on angioplasty of CVS using stent retrievers indicate favorable results for this treatment method. The success rates were defined by radiographic resolution of vasospasm and ranged from 82.1% to 100%. Furthermore, no retreatment of recurrent CVS was required in the same or following sessions, with the exception of three patients in the Kwon et al. study, who were treated first with medication and then with SA [18].

Our results reveal a lower efficacy rate when CVS is treated by SA using a stent retriever or pRELAX. A total of 55.1% of the treated segments showed radiographic resolution of vasospasm, and 40.8% required a different EVT method in the same treatment session or additional sessions on the following days due to recurrent CVS. Our experience has shown that in many cases SA with a stent retriever or pRELAX does not provide sufficient dilatation of the vessels affected by CVS and does not lead to the desired induced vascular paralysis after angioplasty (i.e., the expected long-term effect).

The concept of treating post-aSAH CVS using the Stent-ReLACSS arose from incidental experience during the management of a periprocedural complication. One of the co-authors (TL) encountered a thromboembolic complication during the diagnostic cerebral angiography of a patient with an aSAH. The patient was treated with mechanical thrombectomy (mTE) and underwent microsurgical clipping of the ruptured aneurysm. During the following postoperative phase severe CVS developed, which did not affect the vessel which had previously undergone mTE.

Despite our limited experience with CVS treatment using the pRELAX RM, this method demonstrated radiographic resolution of vasospasm, no periprocedural complications, and no recurrent CVS in any of the treated vascular segments of the ICA and MCA. None of the treated vessel segments required retreatment in the same session or in subsequent sessions. The treated segments showed regular vessel diameters in the angiographic controls throughout the in-hospital period. Postprocedural CT scans showed no new territory or major ischemia in the territory supplied by the treated segments. Microemboli were not considered or evaluated in this study due to the severe clinical conditions of the enrolled CVS patients.

Vasospastic vessels usually require mechanical vasospasmolysis to become paralyzed and dilated. When considering the effects of CVS treatment, it is important to remember Poisseuille’s Law, which states that doubling the radius of a vessel increases its flow rate by a factor of 16. Balloon dilatation is a viable option but the risk of vessel dissection and even rupture, as well as interruption of blood flow and difficulty in distal vessel delivery, remain problematic. Vessels with CVS are predisposed to paralysis after mechanical vasospasmolysis and can therefore be treated with devices with lower radial force [18]. Traction of the pRELAX device through the vasospastic vessels causes damage to the underlying smooth muscle cells and the endothelium, resulting in long-term paralysis of the vessels affected by CVS.

Similarly, the Stent-ReLACSS offers benefits similar to stent angioplasty compared with vasodilators or balloon angioplasty for the treatment of CVS:

- No flow arrest.
- Ability to track stents into distal vessel segments.
- Familiarity with the use of stents from mechanical thrombectomy.
- A likely lower risk of perforation than balloon angioplasty because of operator independent stent expansion.
- Long-lasting vasodilation.
- Short procedure time.
- Ability to inject vasodilating drugs at the same time.

Neurointerventional research in the last 30 years has led to tremendous improvements in the treatment of many endovascular emergencies such as stroke and aSAH. These treatments have resulted in a dramatic reduction in morbidity and mortality rates; however, CVS remains the major cause of poor clinical outcomes after aSAH. Neither medicinal vasospasmolysis nor balloon angioplasty have changed this situation, largely due to lack of long-term results and significant complication rates. Stent angioplasty with stent retrievers, pRELAX, or Comaneci devices has also failed to provide relevant improvement in long-term outcomes. Repeated endovascular treatment for recurrent CVS increases the risk of complications [37] and may cause respiratory and cardiovascular complications due to the difficult management of aSAH patients.

The Stent-ReLACSS may seem relatively aggressive; however, the procedure is similar to mechanical thrombectomy for ischemic stroke, which is performed hundreds of times daily worldwide. In our first experience, this method represents the long-awaited method for CVS treatment because of its long-term results and has no periprocedural and postprocedural complications. The pRELAX was our first choice for the Stent-ReLACSS due to its ability to provide constant high radial force over the entire working length of the device; however, due to its large profile and high radial force, the device was only used in the ICA and MCA up to the M2 segment. Treatment through the BA, PCA, and ACA was avoided due to our lack of long-term experience applying the method in the distal vessel segments. The pRELAX requires a 0.021″ ID microcatheter, which allows atraumatic navigation to the distal M2 segment, and the device is designed with atraumatic distal ends for safe device placement.

### Limitations

The Stent-ReLACSS for the treatment of CVS and the present study has the following limitations:

- The currently available version of the pRELAX requires an 0.021″ ID microcatheter, and catheterization of very narrow brain arteries with such a catheter can be challenging.
- The Stent-ReLACSS has only been evaluated for treating CVS of the MCA and ICA after aSAH.
- The data presented here come from anecdotal cases treated under emergency conditions according to the knowledge and experience of the senior author.
- A prospective registry addressing the safety and efficacy of Stent-ReLACSS is warranted.

## Conclusion

Our initial experience with Stent-ReLACSS has revealed several potential advantages over the use of vasodilators, balloon angioplasty, or stent angioplasty. This method is efficient and safe, and it may indeed constitute a successful treatment strategy for CVS following aSAH, which has been highly demanded for some time. Nevertheless, the development of a device with a reduced crossing profile for the treatment of more distal and smaller caliber vessels with CVS using traction maneuvers should be encouraged. A randomized controlled trial comparing the prophylactic application of Stent-ReLACSS with best medical treatment (e.g., calcium channel blockers) in high-risk patients for CVS is in preparation and might provide new options for the treatment CVS after aSAH.

## Data Availability

The entire data as well as case documentation are available in anonymous form from the senior author upon reasonable request.
